# Revisiting *Campylobacter jejuni* Virulence and Fitness Factors: Role in Sensing, Adapting, and Competing

**DOI:** 10.3389/fcimb.2020.607704

**Published:** 2021-02-03

**Authors:** Abdi Elmi, Fauzy Nasher, Nick Dorrell, Brendan Wren, Ozan Gundogdu

**Affiliations:** Faculty of Infectious and Tropical Diseases, London School of Hygiene and Tropical Medicine, London, United Kingdom

**Keywords:** *Campylobacter jejuni*, virulence, host-pathogen, sensing, adaptation, stress and survival

## Abstract

*Campylobacter jejuni* is the leading cause of bacterial foodborne gastroenteritis world wide and represents a major public health concern. Over the past two decades, significant progress in functional genomics, proteomics, enzymatic-based virulence profiling (EBVP), and the cellular biology of *C. jejuni* have improved our basic understanding of this important pathogen. We review key advances in our understanding of the multitude of emerging virulence factors that influence the outcome of *C. jejuni*–mediated infections. We highlight, the spatial and temporal dynamics of factors that promote *C. jejuni* to sense, adapt and survive in multiple hosts. Finally, we propose cohesive research directions to obtain a comprehensive understanding of *C. jejuni* virulence mechanisms.

## Introduction

**Figure 1:**
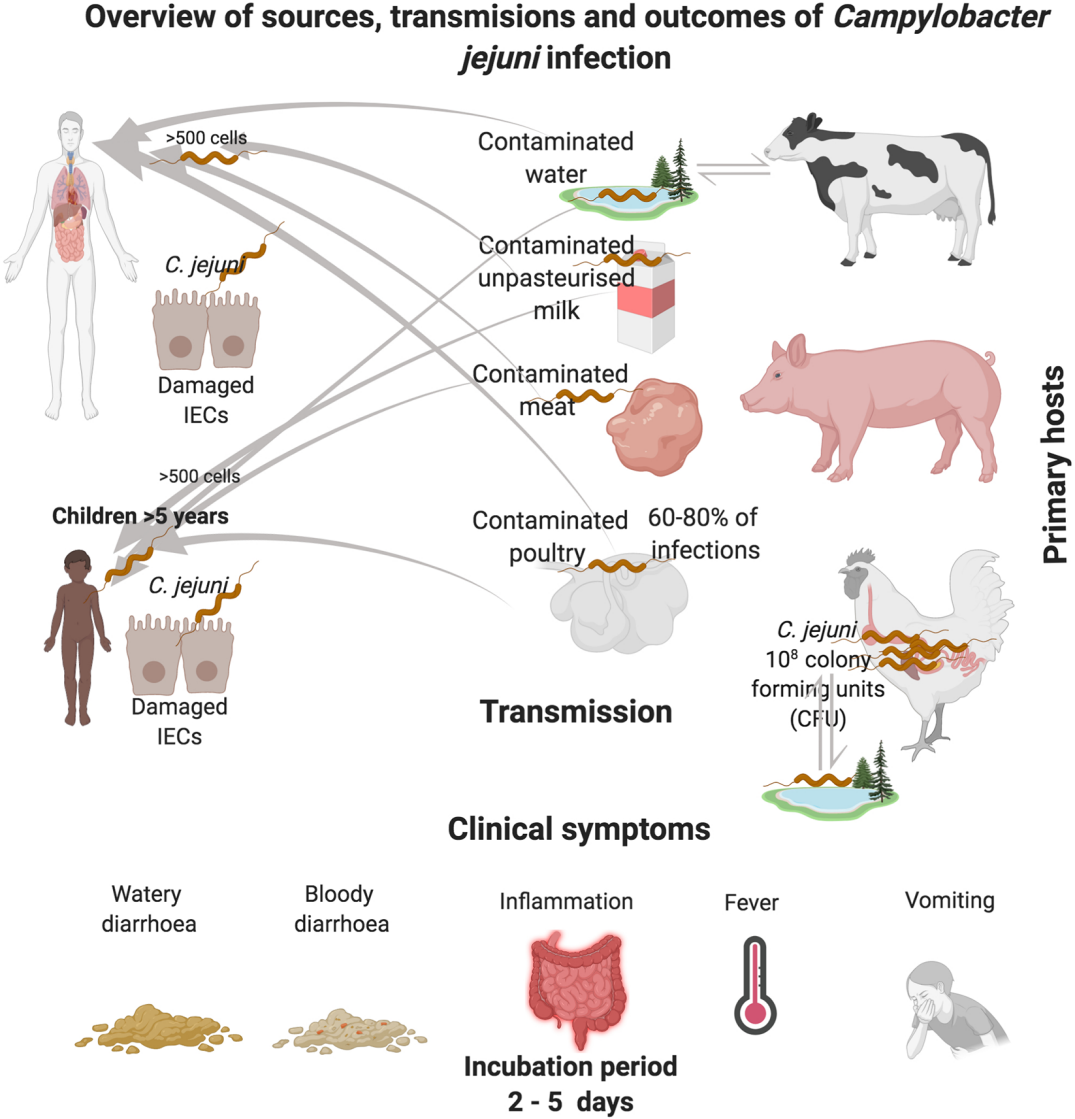
Overview of *C. jejuni* reservoirs and transmission routes of infection. *C. jejuni* reside in the GI tract of chickens, where the bacteria can be spread through consumption of contaminated poultry products. *C. jejuni* transmission can also occur *via* the consumption of contaminated raw cows drinking milk (RDM) which can occur during the milking process, most commonly *via* fecal contamination of udders. Pigs are also recognized as reservoirs of *C. jejuni*. Contamination of the environment can also occur *via* host fecal contamination. *C. jejuni* can persist for long periods in feces, milk and water, especially at temperatures close to 4℃. In adverse conditions, *C jejuni* converts to a viable nonculturable form that can be reactivated when ingested.

*Campylobacters* are the leading cause of bacterial foodborne gastroenteritis in the world. There are 31 different species^1^ and 10 sub-species within the genus *Campylobacter* (Garcia-Sanchez et al., 2018; Wilkinson et al., 2018). The *Campylobacter* genus encompasses several clinically relevant species, such as *Campylobacter jejuni* subsp. *jejuni*, *Campylobacter coli*, *Campylobacter fetus*, *Campylobacter lari*, *and Campylobacter upsaliensis* (Kaakoush et al., 2015; Garcia-Sanchez et al., 2018). This review focuses on *C. jejuni* subsp. *jejuni* which is the most relevant clinically (Skirrow, 1977; Skirrow, 2006). *C. jejuni* is responsible for 80%–90% of the diagnosed cases of *Campylobacter* infections (Facciola et al., 2017). *C. jejuni* colonizes the gastrointestinal (GI) tract of a wide variety of food-producing animals such as poultry, cattle, sheep and swine (**Figure 1**). However, poultry, particularly chickens are the major source of human infection (Humphrey et al., 2014; Ijaz et al., 2018; McKenna et al., 2020). Outbreaks of *C. jejuni* infections are also associated with exposure to contaminated soil, unpasteurized milk and untreated water sources (Korlath et al., 1985; Hudson et al., 1999; Bronowski et al., 2014; Artursson et al., 2018). Clinical symptoms of *C. jejuni* infection can be watery or bloody diarrhea accompanied by abdominal cramps, nausea, fever and sometimes vomiting (Blaser, 1997; Hansson et al., 2018; Igwaran and Okoh, 2019). Although *C. jejuni* infection is acute and self-limiting, in a small number of patients (1:1000) post infection sequalae can lead to severe neurological disorders such as Guillain-Barré syndrome (Yuki et al., 1993; Nachamkin et al., 1998; Sheikh et al., 1998a; Sheikh et al., 1998b; Houliston et al., 2011). According to a recent report by the World Health Organization (WHO), *C. jejuni* is responsible for 96 million cases of enteric infection globally each year (Havelaar et al., 2015; Bailey et al., 2018). In the United Kingdom, *C. jejuni* is responsible for more than 700,000 cases, of which 22,000 hospitalisations and more than 100 deaths occur each year (Bronowski et al., 2014; John et al., 2017). The economic burden associated with *C. jejuni* infection in the United Kingdom is estimated to be £1 billion per year (Bronowski et al., 2014). Moreover, in the European Union (EU), *C. jejuni* is responsible for estimated cases of 9 million with an economic burden of around €2.4 billion each year (https://www.efsa.europa.eu/en/topics/topic/campylobacter). According to the United States Centers for Disease Control, *C. jejuni* is responsible for an estimated 1.5 million human infections each year^2^ with a staggering economic burden of between $1.3 to 6.8 billion dollars per year.

*C. jejuni* does not possess classical virulence factors observed in bacterial enteropathogens such as enterotoxigenic *Escherichia coli* and *Salmonella* spp. (Gaytan et al., 2016; Park et al., 2018). However, *C. jejuni* has a complex array of fitness and virulence factors (Crόinín and Backert, 2012; Backert and Hofreuter, 2013; Backert et al., 2013) which aid the bacterium to respond to the defense mounted by the host; *C. jejuni* can adhere, invade and temporarily survive inside human intestinal epithelial cells (IECs) *in vitro*. We review recent progress made in understanding *C. jejuni* pathogenesis. We highlight findings from several approaches that pioneered the integration of selective mutagenesis, phenotypic assays, high-resolution proteomics and ‘omics. Finally, we describe challenges ahead for successful research in understanding how *C. jejuni* causes disease in humans.

## *C. jejuni* Virulence Factors, a Breakthrough in Understanding the Missing Link

In early 2000, the availability of the full genome sequence of *C. jejuni* NCTC 11168, isolated from the feces of a diarrheic patient in 1977 by Martin Skirrow, marked a new era in the study of the pathogenesis of this major enteric pathogen (Skirrow, 1977; Parkhill et al., 2000). The annotation of the full genome sequence revealed the absence of genes encoding for a non-flagellar type 3 protein secretion system (NF-T3SS). This finding has raised an intriguing question: Does *C. jejuni* sense, inject and secrete putative virulence factors into host cells? In contrast to the absence of NF-T3SS, the genome sequence shed light on the presence of a genomic locus encoding a novel bacterial protein *N*-glycosylation (*pgl*) system, absent in other enteropathogens (Szymanski et al., 1999; Linton et al., 2005). This 11 gene locus encodes for all the necessary enzymes for *N-*linked *pgl* system to produce a conserved heptasaccharide consisting of GalNAc–α1,4-GalNAc–α1,4(Glcβ1,3)-GalNAc–α1,4-GalNAc–α1,4-GalNAc–α1,3-Bac (Bac is bacillosamine or 2,4,-diacetamido-2,4,6-trideoxyglucose (Young et al., 2002; Jervis et al., 2012). *C. jejuni* conserved heptasaccharide has been found to modify up to 100 periplasmic and membrane-bound proteins while it also appears to be responsible for multiple cell functions (Cain et al., 2019; Abouelhadid et al., 2019; Abouelhadid et al., 2020). A feature of the availability of *C. jejuni* genome sequence was the identification and characterization of different glycostructures. In addition to the *N*-linked *pgl* system, other studies have facilitated systematic analysis of genes encoding for flagellar biosynthesis and modification (Jagannathan et al., 2001; Hendrixson and DiRita, 2003; Konkel et al., 2004), lipooligosaccharide (LOS) (Parker et al., 2005; Parker et al., 2008; Kanipes et al., 2008; Hameed et al., 2020) and capsule polysaccharide (CPS) (Karlyshev et al., 2001; Karlyshev et al., 2005). In parallel, the genome sequence of *C. jejuni* identified a large repertoire of phase-variable genes (Guerry et al., 2002; Aidley et al., 2018). The genome sequence of *C. jejuni* further accelerated characterization of repertoire of virulence and fitness factors such as putative adhesins (Konkel et al., 2005), proteases (Brondsted et al., 2005), autotransporters (Ashgar et al., 2007), chemotaxis regulatory genes (Marchant et al., 2002) and the cytolethal distending toxin (CDT) (Purdy et al., 2000). Sequencing the genomes of various *C. jejuni* isolates have also elucidated strain-specific genetic diversity, noticeably the finding of the putative pVir plasmid in *C. jejuni* strain 81–176 (Bacon et al., 2000). Because of the high genome plasticity of *C. jejuni*, genome sequencing also facilitated genome-wide association studies (GWAS) which provided insight into the prevalence of *C. jejuni* virulence genes, antimicrobial resistance markers as well as relatedness of human clinical isolates (Sheppard et al., 2013; Buchanan et al., 2017). Understanding the genetic variability of *C. jejuni* isolates is important for defining key factors that contribute to its ability to host adaptation and evolution. Some *C. jejuni* strains are restricted to specific host while there are *C. jejuni* strains with multi-host lineages. Defining how *C. jejuni* adapts to hosts is an enduring challenge. However, study has demonstrated that one factor that is driving rapid *C. jejuni* host adaptation is gain and loss of *panBCD* genes encoding for vitamin B_5_ biosynthesis pathway (Sheppard et al., 2013). Recently, the advent of large scale genome sequencing has also identified *C. jejuni* isolates possessing Type VI Secretion System (T6SS) (Corcionivoschi et al., 2015; Ugarte-Ruiz et al., 2015), offering the potential to better understand the role of T6SS in *C. jejuni* pathogenesis (Liaw et al., 2019).

## *C. jejuni* in the Host-Pathogen Crosstalk: Virulence and Fitness Factors

In its natural environment *C. jejuni* adapts, survives and proliferates in the nutrient-rich mucous layer of the avian GI tract. *C. jejuni* growth in chicken ceca exceeds 10^8^ colony-forming units per g of cecal contents (CFU)/g (Dhillon et al., 2006; Hermans et al., 2011; Gormley et al., 2014). The transition of *C. jejuni* from nutrient-rich chicken ceca to the environment exposes *C. jejuni* to perturbations. These perturbations unveil *C. jejuni* to atmospheric oxygen (ca. 21% O_2_) and temperature fluctuations which thus alter *C. jejuni* nutrient acquisition and metabolism. In the context of human infection, *C. jejuni* faces additional stresses such as peristalsis and expulsion in the GI tract*. C. jejuni* also faces endogenous stresses ranging from oxidative, nitrosative, pH fluctuations and cationic stresses. The ability to persist in spite of various stresses indicate *C. jejuni* harbors complex virulence and fitness factors (Hermans et al., 2012). These virulence and fitness factors do not only confer protection but also play a role in the ability of *C. jejuni* to sense, adapt and compete the constantly changing host microenvironments, working for example as sensors and/signal molecules, adhesins for host receptors, and/or effectors for invasion and intracellular survival.

*C. jejuni* interaction and invasion of human IECs induce numerous downstream host signaling pathways. *C. jejuni* activates mitogen-activated protein kinases (MAPKs), extracellular signal-regulated kinase (ERK) and p38, leading to the induction of a potent pro-inflammatory cytokine interleukin-8 (IL-8) (MacCallum et al., 2005). IL-8 is an important pro-inflammatory cytokine of IECs and acts as a chemotactic factor of immune cells. However, it is hypothesized that induction IL-8 from human IECs which is found to correlate with an increase in circulating neutrophils to the site of infection can inadvertently exacerbate the classical acute inflammatory symptoms. *C. jejuni* induction of Erk and p38 signaling pathways is dependent on bacterial *de novo* protein synthesis and a functional flagellum (Jin et al., 2003; Watson and Galan, 2005).

## *C. jejuni* Flagella: Function and Virulence

*C. jejuni* produces two polar flagella at each pole of the cell, termed as amphitrichous flagellation. *C. jejuni* flagella is a multifunctional organelle which enables the bacterium to avoid hostile environments including forceful peristalsis and expulsion from the GI tract. *C. jejuni* flagella also enable the bacterium to penetrate the viscous mucosa lining of the human IECs, and to reach the distal ileum, jejunum and colon. Thus, *C. jejuni* flagella promotes bacteria motility, chemotaxis and avian colonization. Besides mediating these virulence attributes, *C. jejuni* flagellar also promotes adhesion and invasion into human IECs *in vitro* (Black et al., 1988; Grant et al., 1993; Szymanski et al., 1995; Konkel et al., 1999), biofilm formation (Svensson et al., 2014) and non-flagella protein export. The latter enables *C. jejuni* to secrete ∼18 putative virulence-associated proteins termed *Campylobacter* invasion antigens (Cia) (Konkel et al., 2004; Christensen et al., 2009). Some of *C. jejuni* Cia proteins are required for invading human IECs *in vitro*, for instance CiaC plays a role in invasion whereas CiaI is required for intracellular survival in human IECs (Buelow et al., 2011; Neal-McKinney and Konkel, 2012). Interestingly study showed CiaD involves in maximal activation of the MAP kinase signaling pathways Erk 1/2 and p38 resulting in the secretion of IL-8 (Samuelson et al., 2013).

*C. jejuni* flagella synthesis and glycan modification involves over 50 flagellum-related genes. The flagellum is composed of three major parts, the basal body, which crosses the bacterial cell membrane, as well as a flagellar-associated cytoplasmic ring, the hook complex and the flagellar filament. Debates had focused on finding relationships between *C. jejuni* flagellum, motility, colonization and secretion. *C. jejuni* flagellar filament contributes to bacterial motility (Wassenaar et al., 1991; Guerry et al., 1991), adherence and colonization. The flagellar filament is composed of subunits of FlaA and FlaB proteins. *C. jejuni* flagellin proteins are *O-*linked glycosylated and the *O*-linked glycosylation is specific to the serine and threonine residues on a flagellin subunit which is modified by pseudaminic acid (Pse) and derivatives containing acetyl and acetamindino groups (PseAcOAc or PseAm, respectively (Thibault et al., 2001; Schirm et al., 2005). Sometimes *C. jejuni* flagellin subunits are modified with legionaminic acid (Leg), moieties (Logan et al., 2009; Schoenhofen et al., 2009; Howard et al., 2009). *C. jejuni* flagellar subunit FlaA rather than FlaB is essential for *C. jejuni* motility. This is supported by evidence that showed a mutation of the *flaA* gene led to the generation of non-flagellated and non-motile cells (Nuijten et al., 1990; Wassenaar et al., 1991). By contrast, the mutation of *flaB*, has no impact on *C. jejuni* flagella synthesis and motility. These findings suggest that FlaA protein, rather than motility, is essential for *C. jejuni* optimal colonization in chickens (Wassenaar et al., 1993). However, subsequent studies have identified *C. jejuni* mutant with normal but paralyzed flagella that is also non-motile and had a reduced ability to colonize chickens (Yao et al., 1994). The role *of C. jejuni* flagella in chicken colonization is further confirmed through mutation of the flagellar motor genes *MotA* and *MotB* which are essential for the rotation of the flagella. A *motAB* mutant produced non-motile cells with a full-length flagellum that is unable to rotate, thus unable to colonize chickens (Hendrixson and DiRita, 2004). Other *C. jejuni* flagella genes that have been studied include the flagellar sigma factor σ^28^ (*fliA*) and the alternative sigma factor σ^54^ (*rpoN*). These two sigma factors regulate a large number of genes that are responsible for the expression and function of *C. jejuni* flagella. For example, sigma σ^28^ is known to regulate the major flagellin gene *flaA* and some other late flagellar genes which control synthesis of proteins forming motor and chemotaxis proteins. On the other hand, *C. jejuni* σ^54^ involves the transcription of genes encoding for the hook, basal body, and minor flagellin *flaB*. In the context of host colonization and infection, mutation of σ^54^ (*rpoN)* gene results a non-motile cells that are unable to colonize chickens (Fernando et al., 2007), adhere to and invade into human IECs *in vitro* (Wassenaar et al., 1991). Also, *C. jejuni* flagellar functions as an organelle to secrete flagellar co-expressed determinants (Feds) which are required for efficient invasion of human IECs *in vitro* (Song et al., 2004; Barrero-Tobon and Hendrixson, 2012). A unique feature of *C. jejuni* flagellar filament is its mechanism to escape immune interaction with Toll-like receptor 5 (TLR5). TLR5s are found at the basolateral side of the human IECs and recognize a highly conserved epitope in bacterial flagellin. However, *C. jejuni* flagellar filament evades TLR5 activation because it fails to make complementary contacts with the TLR5 LRR9 loop (Song et al., 2017). This is attributed to sequence divergence of *C. jejuni* flagellin particularly the highly conserved epitope found in most γ-proteobacteria and Firmicutes bacterial flagellin. Recently, specific amino acids found in *C. jejuni* flagellar filament have been shown to mediate weakened binding to human TLR5 (Kreutzberger et al., 2020).

## *C. jejuni* Capsular Polysaccharide (CPS)

The first evidence of a CPS at the surface of *C. jejuni* was reported in 2001 (Karlyshev et al., 2001). *C. jejuni* CPS is found on the outermost layer of the cell surface of the bacterium and it is composed of a rare structure of diverse repeating units of sugars (Karlyshev et al., 2005; McNally et al., 2005; Gilbert et al., 2007). *C. jejuni* CPS possess a heptoses sugar with an unusual configuration (e.g., ido, gulo, and altro) and nonstoichiometric modifications on the sugars, including ethanolamine, aminoglycerol, and O-methyl phosphoramidate (MeOPN). Unsurprisingly, *C. jejuni* CPS is the major sero-determinant of the Penner serotyping scheme of *C. jejuni *strains (Karlyshev et al., 2000). Currently, there are more than 47 different *C. jejuni* Penner serotypes of the bacterial CPS with some forming related serotype complexes (Poly et al., 2015). The structural variations of *C. jejuni* CPS reflects differences in the genetic content of the genomic locus that drives CPS biosynthesis (Karlyshev et al., 2005). *C. jejuni* CPS contains homopolymeric tracts which are prone to phase variation. As expected, homopolymeric tracts allow a rapid on/off switching of the *C. jejuni* CPS genes resulting in variations in CPS arrangements even in *C. jejuni* isolates that have identical gene contents. In addition to the phase variation observed in CPS sugar composition, *C. jejuni* CPS is also modified with ethanolamine, glycerol, and nonstoichiometric MeOPN modifications in approximately 75% of *C. jejuni* strains (Thota et al., 2018).

*C. jejuni* CPS plays a role in bacteria pathogenicity (Guerry et al., 2012; Bolton, 2015). *C. jejuni* CPS is required to resist complement-mediated killing (Bacon et al., 2001; Keo et al., 2011), invade into human IECs *in vitro* (Bachtiar et al., 2007; Corcionivoschi et al., 2009), colonization of chickens (Jones et al., 2004), and diarrheal disease in ferrets (Bacon et al., 2001). Consistently, the nonstoichiometric modification of CPS with MeOPN has also been demonstrated to be essential for complement resistance. The role of CPS in *C. jejuni* resistance to complement-mediated killing is supported by evidence showing *C. jejuni* expressing full CPS structure but lacking MeOPN, displayed the same pattern of serum killing as a nonencapsulated *kpsM* mutant, which lacked CPS. Also, study, using *Galeria mellonella* larvae infection model demonstrated *C. jejuni* expressing full CPS but lacking specific MeOPN modification to be significantly attenuated in virulence (Champion et al., 2010). This same study suggested the structure of the MeOPN moiety has a remarkable similarities to the active structures of organophosphorous pesticides (McNally et al., 2007), therefore, the virulence attenuation of *C. jejuni* expressing full CPS but lacking specific MeOPN may be due to a consequence of toxicity provided by the MeOPN. However, from virulence perspective, the role of *C. jejuni* CPS in serum resistance is still unclear as *C. jejuni* induces human β-defensins 2 and 3 (hBD2 and hBD3) from human IECs *in vitro* (Zilbauer et al., 2005).

## *C. jejuni* Putative Adhesins

Adhesins play an important role in the pathogenesis of bacteria to adhere, colonize, and invade into hosts. *C. jejuni* adherence to human IECs *in vitro* involves putative adhesins decorated on its outer membrane (OM) surface. *C. jejuni* adhesins seem to have alternate primary functions, yet some can target the same host receptor such as fibronectin. Once *C. jejuni* adheres to fibronectin on the basolateral side of human IECs, it is preceded by secondary steps that orchestrate cellular invasion (Konkel et al., 2020). The most highly investigated adhesins in *C. jejuni* that exist almost in mutually exclusive fashion are *Campylobacter* adhesion to fibronectin (CadF) and fibronectin-like protein A (FlpA). *C. jejuni* adhesins (CadF and FlpA) are highly conserved among *C. jejuni* strains. CadF and FlpA proteins are important for *C. jejuni* adherence to human IECs and colonization of chickens (Konkel et al., 2020). A *C. jejuni* *cadF* mutant displays reduced ability to adhere to human IECs and chicken hepatoma cell line, LMH cells. *C. jejuni* *cadF* mutant is also unable to adhere to immobilized fibronectin (Talukdar et al., 2020). *C. jejuni* FlpA also promotes *C. jejuni* adherence to human IECs *in vitro* and plays a role in *C. jejuni* colonization of chickens (Flanagan et al., 2009; Konkel et al., 2010; Larson et al., 2013). There are additional *C. jejuni* surface-exposed adhesins, such as *Campylobacter* adhesion protein A (CapA), PEB1 (Kervella et al., 1993; Pei et al., 1998) and PEB4 (Asakura et al., 2007). These adhesins which also play a role in *C. jejuni* adherence to human and chicken IECs *in vitro* represent the multifactorial ability of *C. jejuni* virulence mechanisms. However, study suggested that PEB1 is not required for adhering to chicken LMH cells but rather as a transporter of amino acids aspartate and glutamate (Leon-Kempis Mdel et al., 2006). Unfortunately, an important gap in our current knowledge is the lack of mechanistic insight as to how *C. jejuni* orchestrates adherence steps to IECs. This is due in part to the fact that some of the adhesins identified to date display an overlap in binding mechanisms, a factor that confounds straightforward analysis of *C. jejuni* adhesion mechanisms. It is hypothesized that these *C. jejuni* different adhesins are required in the multiple steps of infection. First, to adhere to the mucosal layer at the luminal side of human IECs and then to adhere to the fibronectin receptor at the basolateral side of IECs.

## Other *C. jejuni* Outer Membrane Channels

*C. jejuni* produces numerous virulence and/or fitness proteins that function as major outer membrane proteins (MOMPs). Two of the most well characterized MOMPs in *C. jejuni* are MOMP and OMP50. *C. jejuni* MOMP is also referred to as PorA. In contrast to *E. coli*, *C. jejuni* possesses only one MOMP that is present in all isolates and is highly (but not absolutely) conserved in other *Campylobacters* (Ferrara et al., 2016). *C. jejuni*, MOMP, is a 44-kDa protein, with sequence signature typical of β-barrel porin seen in other enteropathogens (Amako et al., 1996; Ferrara et al., 2016). *C. jejuni*, MOMP is relatively well characterized compared to OMP50. As might be expected, considering its association with the outer surface of the bacterial cell, *C. jejuni* MOMP exhibits substrate selectivity and functions as a control channel for the entry/exit of nutrients and other specific molecules (Dhanasekar et al., 2017). Mutation of *porA* have been thought to be lethal due to critical structural and transport functions. However, inactivation of *porA* enhances sensitivity to certain hydrophilic antibiotics (Iovine, 2013). Unlike MOMP, which is present in most *Campylobacter*s, Omp50 is only found in *C. jejuni* and *C. lari* strains, but not in *C. coli* (Dedieu et al., 2008). The synthesis of Omp50 is tightly regulated by the host microenvironment. For example, *C. jejuni Omp50* is down-regulated in chicken cecum and up-regulated in rabbit ileal loop (Stintzi et al., 2005; Woodall et al., 2005). Mutation of *Omp50* substantially reduced *C. jejuni* motility and invasion, while it also involves bacterium decreased Nox1-dependent ROS generation (Corcionivoschi et al., 2012).

## *C. jejuni* Putative Proteases: New Perspective in Virulence Involvement

Recent characterization of *C. jejuni* putative proteases represent an important step forward in the efforts to dissect *C. jejuni* pathogenesis. As opposed to traditional candidate-mutant experimental approaches, a proteomics analysis coupled with enzymatic-based virulence profiling (EBVP) have shed light on the specific role of *C. jejuni* putative proteases in adhesion to and invasion into human IECs *in vitro*. *C. jejuni* secretes outer membrane vesicles (OMVs) that contain three active serine proteases (HtrA, Cj0511, and Cj1365c) (Elmi et al., 2012). The mechanism responsible for the abundance of these serine proteases in OMVs remains elusive. However, *C. jejuni* proteases have been demonstrated to contribute targeted damage to human IECs *in vitro* (Elmi et al., 2016). Treatment of human IECs with active protease result in cleavage of IECs tight and adherens junction proteins, namely occludin and E‐cadherin. The targeted proteolytic activity of *C. jejuni* OMVs also enhance *C. jejuni* adhesion to and invasion into IECs *in vitro* (Elmi et al., 2016). Moreover, follow-up study has shown that bile salt sodium taurocholate (ST) upregulates *C. jejuni* expression of *htrA*, *Cj0511*, *Cj1365*, and the *cdtABC* operon, highlighting the importance of bacterium adaptation to host metabolites (Elmi et al., 2018). Furthermore, recent study has demonstrated that physiological concentrations of ST regulates *C. jejuni* OMVs production through changes in expression of the maintenance of lipid asymmetry (MLA) pathway (Davies et al., 2019). Although most of the examples discussed above had focused on the role of serine proteases in virulence, it should be remembered that *C. jejuni* OMVs also contain a cocktail of virulence and fitness factors, including stress response enzymes, adhesins, CDT, lipoproteins and other metalloproteases, which also play an important role in bacterial virulence. Thus, suggestions have been raised that *C. jejuni* OMVs might also function as fitness and survival factors, allowing the bacterium to adapt new niches, adhere to surfaces, translocate rapidly across IECs, and resist antibiotics and other deleterious circumstances.

## *C. jejuni* Fitness and Virulence Factors: Role in Stress Adaptation, Temperature, Nutrient Sensing, and Metabolic Rewiring

As *C. jejuni* transitions from nutritionally rich ceca in the GI tract of chickens to accidentally infect humans, the bacterium faces formidable stresses. Here, the term “stress” refers to environmental and human host stresses that reduce *C. jejuni* fitness or negatively impact on its virulence. Unlike other entero-pathogens, *C. jejuni* does not have homologs of the classical stress response regulators such as SoxRS and OxyR found in *E. coli* and *Salmonella* spp. respectively. SoxRS regulates response to redox-active compounds while OxyR responds to hydrogen peroxide (Nunoshiba et al., 1992; Zheng et al., 1998). In addition, *C. jejuni* lacks transcription regulators such as cold shock protein A (CspA) and leucine-responsive regulatory protein (Lrp) (Calvo and Matthews, 1994; Murphy et al., 2006; Keto-Timonen et al., 2016). Besides, *C. jejuni* does not possess the classical alternative sigma factors such as RpoS (σ^38^) although it has limited sigma factors including RpoD (σ^70^), RpoN (σ^54^), and RpoF/FliA (σ^28^). Interestingly, *C. jejuni* possesses unique and yet unresolved mechanisms to survive under various stress conditions. *C. jejuni* utilizes OmpR‐type response regulators such as *Campylobacter* oxidative stress regulator (CosR) (Hwang et al., 2011), peroxide-sensing regulator (PerR) (Palyada et al., 2009) and Multiple Antibiotic Resistance Regulator, MarR‐type regulators designated for response to peroxide stress (Gundogdu et al., 2016). *C. jejuni* CosR is a pleiotropic regulator that controls the expression of genes involved in various cellular processes, especially genes that involve in macromolecule biosynthesis, metabolism, and oxidative stress response (Kim et al., 2015b). The genes that CosR regulates mostly encode for stress response-related proteins such as the DNA binding protein from starved cells (Dps), rubredoxin oxidoreductase/rubrerythrin (Rrc), alkyl hydroperoxide reductase (AhpC), and superoxide dismutase (SodB). On the other hand PerR, non-OxyR-dependent regulator, controls transcription of peroxide as well as the superoxide defense genes particularly under oxidative stress conditions. For instance, *perR* mutation abrogates the transcriptional response of *ahpC*, *katA*, and *sodB* to oxidants (Kim et al., 2015a).

*C. jejuni* also possesses global transcriptional regulators such as carbon starvation regulator (CsrA), ortholog of the *E. coli* global posttranscriptional regulator CsrA. In addition, *C. jejuni* has two-component regulatory systems (TCRS) such as *Campylobacter* planktonic growth regulator (CprRS) (Svensson et al., 2015; El Abbar et al., 2019). Mutation of *csrA* results in *C. jejuni* cells with altered motility, biofilm formation, adherence to and invasion of human IECs cells and resistance to oxidative stress (Fields and Thompson, 2008). CprRS is two‐component systems regulator typically consisting of a sensor kinase and a response regulator. The CprR response regulator is essential and mutation to the *cprR*, is lethal to *C. jejuni*, but a *cprS* mutation, results in decreased expression of *SodB*, *Rrc* and *LuxS*. *C. jejuni* also possesses a ferric uptake regulator (Fur) to control the expression of a range of oxidative stress genes, to prevent the build-up of toxic levels of iron within the cell (Butcher et al., 2012). In addition to the stress-responsive regulators, *C. jejuni* KatA and SodB proteins play critical roles in detoxification, SodB detoxifies free radicals $O_{2}^{-}$ while KatA contributes for the detoxification of H_2_O_2_ (Atack and Kelly, 2009). SodB also contributes to *C. jejuni* chicken colonization and intracellular survival in human IECs *in vitro* (Palyada et al., 2009; Novik et al., 2010). *C. jejuni* cell surface structures such as flagella, CPS, LOS and OM also can act at the interface between the bacterium and the extracellular environment. These cellular surface structures assist *C. jejuni* to sense environmental and host stresses, in principle, inducing a collective response to protect the bacterium from damage caused by stresses.

### Environmental Stress Survival and Adaptation

In light of its relatively small genome (1.6–1.7 Mb), it remains enigmatic how *C. jejuni* senses, adapts and persists in diverse environmental stresses. *C. jejuni* requires optimal oxygen concentrations of approximately 5%–10% for growth; however, the bacterium can survive in the environment, which is rich in oxygen (ca. 21% O_2_). This variation in oxygen concentration constraints *C. jejuni* to rewire its physiology to adapt flexible metabolic pathways. The requirement of 5%–10% O_2_ for growth is governed by single class I-type Ribonucleotide Reductase (RNR) (Burnham and Hendrixson, 2018). This is an oxygen-dependent enzyme that catalyses the *de novo* conversion of ribonucleotides diphosphates (NDPs) to deoxyribonucleotides diphosphates (dNDPs), and therefore plays a pivotal role in maintaining *C. jejuni* synthesis of deoxyribonucleotide (dNTP). Besides, *C. jejuni* also possesses a highly-branched respiratory chain feature that facilitates the use of oxygen as an electron acceptor for one of two respiratory oxidases, cytochrome c oxidase (CcoNOQP), a cbb3-type cytochrome c oxidoreductase and a bd-type (CioAB or CydAB) quinol oxidase (Guccione et al., 2017; van der Stel and Wosten, 2019). The sensitivity of *C. jejuni* pyruvate: acceptor oxidoreductase (POR) and the TCA cycle 2-oxoglutarate: acceptor oxidoreductase (OOR) to oxygen has been suggested as one of the explanations of the so-called ‘*C. jejuni*-oxygen paradox’ - that is, why *C. jejuni* is unable to proliferate in aerobic environment. Also, atmospheric levels of oxygen inactivate *C. jejuni* L-serine dehydratase (SdaA), which catalyses the deamination of serine and converts serine into pyruvate which is further converted to acetyl CoA, which is oxidized *via* the TCA cycle to carbon dioxide and free energy. SdaA is essential for colonization of the avian gut (Velayudhan et al., 2004). The ability of *C. jejuni* to tolerate oxygen in the environment can also vary between strains. For instance, study has found a higher prevalence of some strain genotypes in environmental samples attributing these variations in oxygen tolerance (Champion et al., 2005; Bronowski et al., 2014). Besides, another study has reported atypical *C. jejuni* Bf strain that is oxygen tolerant (Rodrigues et al., 2015; Bronnec et al., 2016a). This strain has been demonstrated to have protective mechanisms against oxidative stress which is thought to be mediated by regulation of genes involved in oxidative stress response and biofilm formation (Bronnec et al., 2016b). Interestingly, recent assessment of *C. jejuni* phospholipidome profile has indicated that *C. jejuni* phospholipidome have an unusually high percentage of lysophospholipid. Lysophospholipids are small bioactive lipid molecules characterized by a single carbon chain and a polar head group. It is hypothesized lysophospholipid allows *C. jejuni* to be motile under low O_2_ conditions (Cao et al., 2020a). This is a significant observation considering the requirement of *C. jejuni* to adapt to the low oxygen deep in the mucus layer of the human GI tract. This could give *C. jejuni* competitive advantage when competing with other microbiota that colonize the mucosal layer as it transitions into the IECs. In addition, the ability of *C. jejuni* to sense environmental oxygen have been thought to correlate altering its membrane lipid composition which could be crucial for biofilm formation.

### *C. jejuni* Biofilm: Environmental Adaptation and Persister Phenomena

*C. jejuni* adaptation to an oxygen-rich environment such as contaminated freshwater, poultry meat or raw milk can be attributed to the ability of the bacterium to form biofilms on different substrates. *C. jejuni* can attach and persist on a variety of abiotic and biotic surfaces, and several studies have reported on the viable but non-culturable (VBNC) state (Teh et al., 2014; Magajna and Schraft, 2015). *C. jejuni* cells switch to VBNC state to survive better under adverse environmental conditions. In the environments, *C. jejuni* is exposed to high oxygen tension, limited nutrient availability, heat, acidic pH, temperatures fluctuations and antimicrobials. These environmental constraints are known to stimulate increased *C. jejuni* biofilm formation to a relatively high level, supporting the proposal that *C. jejuni* forms biofilm as a survival strategy outside of the avian host. *C. jejuni* forms increased biofilm in oxygen-rich conditions compared to microaerobic conditions (Reuter et al., 2010). It is commonly agreed that all *C. jejuni* strains form biofilm, however, the ability of *C. jejuni* to form biofilm appears to be strain-dependent (Melo et al., 2017). Interestingly, *C. jejuni* mutant strains deficient in genes encoding for key oxidative stress resistance enzymes such as alkyl hydroperoxide reductase (AhpC) or *C. jejuni*’s sole catalase enzyme (KatA) have been shown to have an increased ability to form biofilm (Oh and Jeon, 2014). This is attributed to the accumulation of reactive oxygen species (ROS) which may serve as a trigger to increase the level of biofilm formed in response to increased oxidative stress. Overexpression of *ahpC* is correlated with decreased biofilm formation, and treatment of the *ahpC* mutant with antioxidants reduces biofilm formation (Oh and Jeon, 2014). *C. jejuni* lacks the classical two-component regulatory systems involved in biofilm formation found in other bacteria, such as GacSA in *Pseudomonas aeruginosa*, however, *C. jejuni* biofilm formation is thought to be under the control of a complex array of regulatory factors that respond to a variety of environmental signals. These complex regulatory factors include global regulator CsrA, *Campylobacter* oxidative stress regulator (CosR), stringent response regulator (SpotT) and CprRS, which have been shown to play an important role in biofilm formation in *C. jejuni* under aerobic conditions (Gaynor et al., 2005; Fields and Thompson, 2008; Svensson et al., 2015; El Abbar et al., 2019). Mutations of *cosR*, *cprRS*, and, *spotT* increase biofilm formation under aerobic conditions, while mutation of the gene encoding for global regulator (CsrA) decreases the ability of *C. jejuni* to form biofilms when grown in static culture as well as increased sensitivity to oxidative stresses (Fields and Thompson, 2008). Interestingly, in other enteric bacteria *spoT* mutation decreases biofilm formation (He et al., 2012). In *C. jejuni*, the mutation of *spoT* alters the expression of genes related to redox balance, metabolism, energy production, and conversion pathways while CosR, a key orphan regulator in the maturation of biofilm, has also been shown to affect the expression of the antimicrobial efflux pump CmeABC (Turonova et al., 2015). CprRS is two‐component systems regulator typically consisting of a sensor kinase and a response regulator. The CprR response regulator is essential and deletion of the cprS sensor kinase enhances biofilms. Current evidence suggests that CprRS likely regulates genes related to aspects of the *C. jejuni* surface structures (Svensson et al., 2015). The molecular mechanism of *C. jejuni* biofilm formation also appears to indirectly correlate with factors required for fitness and virulence. For instance, mutation of the flagella genes *flaA*, *flaB* and the cell surface modification genes *pgp1* and *waaF* have been shown to increase biofilm formation (Reeser et al., 2007). This indicates that *C. jejuni* increases biofilm formation as a survival strategy during stress. Interestingly, a recent study suggests *C. jejuni* does not form biofilms under conditions encountered in the environment but attaches to surfaces or biofilms of other species (Teh et al., 2014; Teh et al., 2019). This is an attractive proposal supporting the notion that *C. jejuni* is a poor biofilm initiator, and is likely to form enhanced biofilms in a “mixed-species biofilm” with other bacteria such as *P. aeruginosa*, *Enterococcus faecalis* and *Staphylococcus simulans*.

### *C. jejuni* Temperature Stress Adaptation

Temperature is a prominent signal used by many enteric pathogens. The strategies enteric pathogens use to sense temperature variation across space, hosts and time broadly acts as a mechanism to adjust bacterial survival and virulence. For *C. jejuni*, the transition from its primary chicken host (42°C) to the environment, the bacterium experiences temperature variation. This temperature variation confines proliferation and shifts *C. jejuni* physiology forcing the bacterium to coordinate fitness and virulence regulatory systems. It is puzzling that *C. jejuni* lacks classical RpoS homolog (Parkhill et al., 2000) and cold shock proteins (Oh et al., 2019), yet *C. jejuni* has the ability to survive in low and/or high nonpermissive temperature growth conditions before reaching human host. *C. jejuni* doesn’t also grow temperatures below ~ 30°C, however the bacterium survives temperature growth range between 4°C to 33°C (Hazeleger et al., 1998). *C. jejuni* survives better at 4°C in various biological milieu than at 25°C (Murphy et al., 2006). *C. jejuni* also survives in water, at low temperatures, for up to 4 months (Oberheim et al., 2020). The ability of *C. jejuni* to survive in cold temperatures is different among strains, with *C. jejuni* strains isolated from human infection being significantly more capable of prolonged survival at 4°C than poultry‐derived strains (Chan et al., 2001). Intriguingly *C. jejuni* also survives extreme freezing temperatures (−20°C) for several weeks (Bhaduri and Cottrell, 2004).

*C. jejuni* genes associated with oxygen tolerance, starvation and osmotic stress are essential for the bacterium to survive in the low temperature. This perplexing physiology of *C. jejuni* seems to be the bottleneck to the efforts aimed to eradicate the risk of *C. jejuni* to human health. The ability of *C. jejuni* to rapidly sense and adapt to cold temperature is largely driven at the transcriptional level (Bronowski et al., 2017). Studies focusing on human infections, use *in vitro* human IECs grown at 37°C to mimic the temperature that the bacteria encounters inside human host. *C. jejuni* ability to sense 37°C is crucial to optimize its fitness and adjust expression of its virulence genes. *C. jejuni* is more invasive into human IECs cultured at 37°C than IECs cultures at 42°C (Aroori et al., 2013). Although the exact mechanism of *C. jejuni* response to temperature stress is not yet explicitly known, changes in temperature are known to affect expression of bacterial heat shock proteins (HSP). *C. jejuni* possesses two-component regulatory systems (TCSs) such as reduced ability to colonize response regulator (RacRS). RacRS function to assist the bacteria to overcome stresses associated with heat shock response. In addition, *C. jejuni* RacR is required for avian colonization and growth while mutation of *racR* alters the expression of selected proteins in both temperature-dependent and independent manners (Hazeleger et al., 1998; Wouters et al., 2000).

### *C. jejuni* Acid Stress Adaptation

*C. jejuni* grows at optimal pH range of 6.5–7.5, while it is also able to survive pH range as low as 5.5 and as high as 8.5. However, *C. jejuni* encounters acidic conditions either in the environment or within the gut of the various hosts that it colonizes. In the context of human infection, *C. jejuni* survives passage through the stomach, where the concentration of acid is high and the pH ranges 1.5–3.5. The molecular strategies that *C. jejuni* uses to sense, adapt and survive the luminal acid concentration in the stomach upon ingestion and within the phagosomes and phagolysosomes of human IECs is not currently known. However, *C. jejuni* tolerance to human GI tract luminal acid is important for disease development. So far, it is hypothesized *C. jejuni* lacks proteins required for acid tolerance such as urease protein found in *Helicobacter pylori*. However, it is intriguing that with low infectious dose of (500–800 bacteria), *C. jejuni* cells survive the gastric acid of the human stomach and continue down to reach the small intestine. Study has demonstrated some *C. jejuni* strains can survive acid exposure at pH 3.5 and above for up to 30 min (Le et al., 2012). Another study has suggested adaptation of *C. jejuni* to the luminal acid concentration in humans requires genes mediating various cellular processes, including those involved in motility, metabolism, stress response, DNA repair and surface polysaccharide biosynthesis (Reid et al., 2008). For instance, *C. jejuni Rpo*N, a classical flagellar transcriptional regulator, which is historically known to play an important role in motility has been demonstrated to be important for the resistance of *C. jejuni* to various stresses including acid stress. This suggested flagella mediated motility is critical for both initial navigation through the acid environment in the GI tract lumen and mucus layer to IECs attachment. *C. jejuni* adaptation to low pH stress also involved the differential expression of genes involve in respiratory pathways, the upregulation of genes for phosphate transport, and the repression of energy generation and intermediary metabolism genes (Reid et al., 2008). Recent study that investigated acid-stressed adaptation of *C. jejuni* under iron-enriched conditions has shown the capacity of *C. jejuni* to survive acid stress is greatly enhanced in presence of iron (Askoura et al., 2020). However, limited information is available about the role which human host microbiota plays in the pathophysiology of *C. jejuni* adaptation in acidity along the gut, although it is evident that many species of the microbiota are able to generate metabolites that have bearing on the composition of GI tract luminal acidity. For example, lactate which is an organic acid that is found in the upper GI tract of human and avian species can act as a chemoattractant signal of *C. jejuni* (Bernalier-Donadille, 2010; Hofreuter, 2014).

### *C. jejuni* Metabolic Sensing and Adaptation

While, as discussed above, *C. jejuni* has complex stress response mechanisms, its ability to resist stresses overlaps its ability to adapt to different metabolic requirements. *C. jejuni* sequenced strain NCTC11168 lacks the glycolytic enzymes glucokinase (Glk) and phosphofructokinase (PfkA) of the classical Embden-Meyerhof-Parnas (EMP) pathway (Parkhill et al., 2000; Guccione et al., 2008; Hofreuter, 2014). *C. jejuni* was once considered to be non-saccharolytic since *C. jejuni* sequenced strain NCTC11168 lacks genes encoding for the complete pentose phosphate (PPP) or Entner-Doudoroff (ED) pathway. Interestingly, few isolates of *C. jejuni* subsp. *doylei* encode a complete ED pathway which suggests the potential to catabolize glucose (Vegge et al., 2016; Garber et al., 2020). The inability to utilize glucose has necessitated *C. jejuni* to utilize amino acids such as serine, aspartate, glutamate, glutamine, proline and asparagine as carbon and energy sources (Stahl et al., 2012; Hofreuter, 2014; Szymanski, 2015). Most *C. jejuni* strains preferentially use serine, aspartate, glutamate, and proline, although certain *C. jejuni* strains can also utilize asparagine and glutamine (Thompson and Gaynor, 2008; van der Hooft et al., 2018). This unique ability to metabolize only a few amino acids allows the bacterium to utilize efficient strategies to include host nutrients into its anabolic processes, to fuel its metabolic pathways and to support its survival and adaptation in hosts with largely commensalism outcome in avian species or pathogenesis in humans. For instance, *C. jejuni* catabolism of serine and aspartate enhances the ability of the bacterium to colonize the avian gut (Hermans et al., 2011), while a *C. jejuni* mutant that is lacking an oxygen-labile serine dehydratase and unable to catabolize serine is demonstrated to be incapable of colonizing chickens (Velayudhan et al., 2004). Furthermore, *C. jejuni* rewires its metabolic requirements during avian colonization and human infection. *C. jejuni* has the ability to adopt an asaccharolytic lifestyle, likely as a strategy to evade microbiome competition. It is known that certain *C. jejuni* strains metabolize sugars such as L‐fucose (Stahl et al., 2011). These *C. jejuni* strains possess an operon for L‐fucose utilisation which until recently has been known to be limited to some *C. coli* and *C. jejuni* subsp. *doylei* strains. L-fucose acts as a chemoattractant for *C. jejuni* (Dwivedi et al., 2016). Interestingly, *C. jejuni* binds to α1, 2-fucosylated glycans, however the L-fucose catabolism is not essential for *C. jejuni* colonization of avian species (Muraoka and Zhang, 2011; Stahl et al., 2011). Furthermore, *C. jejuni* lacks fucosidase enzyme which is essential for the release of the L-fucose from glycosylated host proteins such as mucin. A study recently demonstrated that *C. jejuni* fucose positive strain utilisation of L-fucose is dependent on the fucosidase activity of the gastrointestinal bacterium *Bacteriodes fragilis* (Garber et al., 2020). This same study also revealed that *C. jejuni* becomes more invasive toward human Caco-2 cells in the presence of an exogenous fucosidases from *B. fragilis*.

Recently, examining the idea of a host nutritional role in *C. jejuni* adaptation and pathogenesis, studies showed that *C. jejuni* senses and utilizes catabolic end products of the intestinal microbiota such as short-chain fatty acids (SCFAs) butyrate and acetate, CO_2_-derived hydrogen carbonate, and free amino acids and di-/or oligopeptides, which are released by microbiota from dietary or endogenous proteins (Gao et al., 2017). The ability of *C. jejuni* to sense SCFAs positively regulates many *C. jejuni* amino acids uptake and catabolism systems that are essential for host colonization. SCFAs are found in abundance in the lower regions of the intestinal tracts of avian species and humans where they play a major role in host physiology through nutritional, regulatory, and immunomodulatory functions. However, in the context of *C. jejuni* avian and human colonization, the abundance of butyrate and acetate in the lower GI tract provides the bacterium with a competitive advantage to thrive in this niche (Burnham and Hendrixson, 2018). A prevailing belief is that *C. jejuni* has the ability to spatially differentiate between sections of the GI tract by sensing the presence of acetate and butyrate, and thereby modifying the transcription of its colonization factors (Goodman et al., 2020). This enables *C. jejuni* to obtain sufficient nutrients and resources to allow for optimal survival and persistence in both avian and human intestinal tracts. *C. jejuni* specifically senses butyrate *via* a noncanonical TCS termed BumSR (Goodman et al., 2020). BumS functions as a phosphatase, *via* a noncanonical mechanism for signal transduction in place of a sensor kinase, to control the activity of the cognate BumR response regulator. BumS phosphorylates BumR in response to the presence of butyrate. *C. jejuni* genes known to be induced after sensing butyrate and acetate include genes encoding for nutrient acquisition systems, energy generation pathways, and colonization factors (Goodman et al., 2020). In addition, acetate which is more abundant in the gut is preferred metabolite for *C. jejuni* once the rate-limiting step of carbohydrate metabolism is surpassed in stationary phase. *C. jejuni* also catabolizes organic acids such as lactate which is abundant in the upper gut of avian hosts (Luethy et al., 2017).

## Conclusions and Future Directions

Recent developments in the understanding of *C. jejuni* pathogenesis have combined several experimental approaches that link the functional characterization of various putative genes. Although this is important, characterizing *C. jejuni* virulence and fitness factors requires an integrative approach. In the future, an ideal experiment should involve the use of single-gene inactivations and phenotypic assays, incorporated with integrative multi-omics approach including, transcriptomics, proteomics and metabolomics. This should reveal comprehensive findings that would contribute to the characterization of *C. jejuni* pathogenesis. This approach will also guide us to re-focus on re-characterization of many *C. jejuni* virulence-associated genes that have not yet been fully characterized. From our perspective, the incorporation of integrative multi-omics and phenotypic assays in *C. jejuni* research promises enormous potential. However, there are many challenges and thus, opportunities for further development of experiments involving multi-omics technology. Also, future studies of *C. jejuni* should include refining, optimisation and normalization of experimental design and protocols that represent ideal settings for *C. jejuni* and host cells, allowing researchers to reproduce data. Unsurprisingly, there are a plethora of *C. jejuni* studies that use experimental approaches that give an insight into the selected role of *C. jejuni* putative virulence associate genes. For instance, in stress survival, adhesion, invasion and intracellular survival, however, few studies provide information about the function of such putative genes. Also, integration of *C. jejuni* virulence characterizations with spatial analysis at the various time point and *C. jejuni* strains variability is needed to improve our understanding of *C. jejuni* pathogenesis.

## Author’s Note

For the purpose of this review, we define a virulence factor as a protein (such as a toxin) or macromolecular structure (such as flagellum) that contribute to the ability of the bacteria to cause disease and a fitness factor as a protein or macromolecular structure that, while not required for virulence, offers a competitive advantage during infection.

## Author Contributions

AE: Conceived and designed the structure of the manuscript; AE Created Figure 1; AE, OG, and FN: Wrote the manuscript; AE, BW, ND, OG, and FN: Read and edited the manuscript. All authors contributed to the article and approved the submitted version.

## Conflict of Interest

The authors declare that the research was conducted in the absence of any commercial or financial relationships that could be construed as a potential conflict of interest.

## Footnotes

1 http://www.bacterio.net/campylobacter.html

2 https://www.cdc.gov/campylobacter/technical.html
